# Advancing antibiotic discovery with bacterial cytological profiling: a high-throughput solution to antimicrobial resistance

**DOI:** 10.3389/fmicb.2025.1536131

**Published:** 2025-02-13

**Authors:** Jhonatan Salgado, James Rayner, Nikola Ojkic

**Affiliations:** School of Biological and Behavioural Sciences, Queen Mary University of London, London, United Kingdom

**Keywords:** antibiotic resistance, bacterial cytological profiling, high-throughput screening, antibiotic mechanism of action, bacterial priority pathogen list, cell segmentation, machine learning, deep learning

## Abstract

Developing new antibiotics poses a significant challenge in the fight against antimicrobial resistance (AMR), a critical global health threat responsible for approximately 5 million deaths annually. Finding new classes of antibiotics that are safe, have acceptable pharmacokinetic properties, and are appropriately active against pathogens is a lengthy and expensive process. Therefore, high-throughput platforms are needed to screen large libraries of synthetic and natural compounds. In this review, we present bacterial cytological profiling (BCP) as a rapid, scalable, and cost-effective method for identifying antibiotic mechanisms of action. Notably, BCP has proven its potential in drug discovery, demonstrated by the identification of the cellular target of spirohexenolide A against methicillin-resistant *Staphylococcus aureus*. We present the application of BCP for different bacterial organisms and different classes of antibiotics and discuss BCP’s advantages, limitations, and potential improvements. Furthermore, we highlight the studies that have utilized BCP to investigate pathogens listed in the Bacterial Priority Pathogens List 2024 and we identify the pathogens whose cytological profiles are missing. We also explore the most recent artificial intelligence and deep learning techniques that could enhance the analysis of data generated by BCP, potentially advancing our understanding of antibiotic resistance mechanisms and the discovery of novel druggable pathways.

## 1 Introduction

The World Health Organization (WHO) has declared antimicrobial resistance (AMR) as one of the most severe global health threats facing humanity. AMR is the ability of a microbe to survive and grow in the presence of a chemical thought to prevent this effectively. It has been estimated that in 2019 alone, antimicrobial resistance killed at least 1.27 million people globally, more deaths than HIV/AIDS or malaria, with 4.95 million deaths associated with AMR (Murray et al., 2022). According to the Centers for Disease Control and Prevention’s Antibiotic Resistance Threats Report (Centers for Disease Control and Prevention (U.S.), 2019), in the United States, over 2.8 million antibiotic-resistant infections appear every year, leading to over 35,000 deaths. Furthermore, AMR has been predicted to lead to a total loss of up to $100 trillion for the global economy by 2050 (O’Neill, 2016). These alarming statistics underscore the urgent need to develop effective therapeutics to combat antimicrobial resistance.

The efforts undertaken in the field of AMR until now have not been enough despite the enormous research effort and inventive therapeutic approaches. Since 1940, antimicrobials have been used widely (Gardner, 1940; Gardner, 1945) and beyond treating infections, antibiotics enabled many modern medical procedures, such as open-heart surgeries, organ transplants, and cancer therapies (Hutchings et al., 2019). Even before 1940 and for about 60 years after, most antibiotics were discovered by culturing microbial samples from soil for compounds already expressed by microbes (Walsh, 2000; Hutchings et al., 2019). However, over the last 20 years, the lipopeptides and the oxazolidinones have been the only two new antibiotic classes created and have been effective only against Gram-positive bacteria (Luepke et al., 2017). The last novel antibiotic class introduced to kill Gram-negative bacteria was the quinolones when nalidixic acid was synthesized in 1962 (Tacconelli et al., 2018). Although recent developments have shown potential against Gram-negative bacteria, such as Zosurabalpin, a new antibiotic that disrupts bacterial lipopolysaccharide (LPS) transport from the inner membrane to the outer membrane (Zampaloni et al., 2024), further advancements in antibiotic discovery are needed. To facilitate the discovery of novel druggable pathways, new high-throughput screens based on Bacterial Cytological Profiling have been developed.

This review emphasizes the use of bacterial cytological profiling (BCP) as a highly effective method for discovering novel antibiotics and rapidly identifying antibiotic targets in a cost-effective manner. BCP initially creates a library that captures the overall profile of bacterial morphological and physiological changes at a single-cell level induced by antibiotics with known mechanisms of action. This profile includes details on bacterial cell shapes and sizes, fluorescent intensities and spatial distribution of DNA, and fluorescent distribution of membrane dyes (Nonejuie et al., 2013; Quach et al., 2016; Samernate et al., 2023). The library is then utilized to classify existing antibiotics based on the specific components of bacterial cells they target and to discover new antibiotics. In this review, we also highlight how BCP is used to expand our quantitative understanding of antibiotic pharmacodynamics and bacterial stress responses, as well as how BCP enhances the development of non-traditional antibacterial strategies such as phage therapies (Deep et al., 2024; Tsunemoto et al., 2023; Birkholz et al., 2024; Thammatinna et al., 2020; Soonthonsrima et al., 2023; Naknaen et al., 2023; Naknaen et al., 2024).

## 2 Antibiotic mechanism of action and antibiotic targets

**Figure 1:**
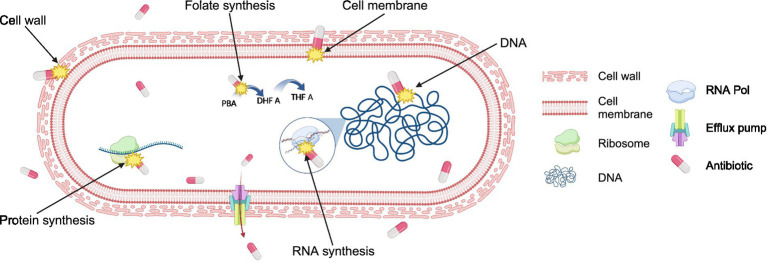
Antibiotic targets in bacteria. Antibiotics typically kill bacteria by targeting at least one of the five major cell components: cell wall, cell membrane, ribosomes, DNA, and RNA. Antibiotics interfere with the synthesis of or the direct damage of cellular structures or components resulting in the inhibition of bacterial growth or irreversible reduction of bacterial cell integrity. Some antibiotics inhibit the synthesis of essential cell components, such as folate synthesis, a precursor for DNA synthesis. Additionally, bacteria have evolved mechanisms such as efflux pumps, which actively transport antibiotics out from the cell, reducing their efficacy and contributing to antibiotic resistance. Figure is created using BioRender.

AMR arises from either genetic alterations or phenotypic changes in pathogens (Corona and Martinez, 2013; Davies and Davies, 2010; Jago et al., 2025). To effectively tackle antibiotic-resistant bacteria, it is essential to understand how antibiotics work, which is known as their mechanism of action (MOA), see Table 1. Understanding MOA involves studying how antibiotics affect bacterial physiology and molecular interaction with bacterial targets (Figure 1). However, identifying the MOA presents a significant limitation in drug discovery. In some cases, the exact MOA is determined years after a drug’s approval, as seen with daptomycin (Grein et al., 2020).

**Table 1:** ^*^General classification of antibiotics based on their target and chemical structure, including their mechanism of action.

Target	Chemical structure	Mechanism of action	Generic name examples
Cell wall	β-Lactams	Inhibit penicillin-binding proteins (PBPs) that crosslink peptidoglycan chains in the bacterial cell wall (Lima et al., 2020), disrupting cell wall integrity and causing cell lysis (Baquero and Levin, 2021).	Penicillins, cephalosporins, cephamycins, carbapenems, and others.
	Glycopeptides	Bind to the acyl-D-Ala-D-Ala terminus of peptidoglycan in Gram-positive bacteria (Reynolds, 1989).	Vancomycin
Membrane	Lipopeptides	Depolarize the cell membrane, reducing the ability to create ATP and inducing cell death (Jerala, 2007).	Daptomycin, Colistin
Fatty acid synthesis	Chlorophenol	Inhibit *fabI,* an enoyl-ACP reductase, blocking the fatty acid synthesis (O’Rourke et al., 2020).	Triclosan
	Oxirane carboxylic acids	Bind to β-ketoacyl-acyl carrier protein synthase, inhibiting fatty acid synthesis. In sterol synthesis, inhibits HMG-CoA synthetase activity (PubChem, n.d.).	Cerulenin
Protein synthesis	Aminoglycosides	Cause mRNA misreading and production of uncompleted proteins by targeting the 30s ribosomal subunit of 16S RNA resulting in cell death (Baquero and Levin, 2021; Davis et al., 1986).	Gentamicin, tobramycin, kanamycin
	Tetracyclines	Bind to 16S rRNA of the 30S ribosomal subunit, inhibiting tRNA binding to 30S and preventing translation (Chopra and Roberts, 2001).	Tetracycline, doxycycline, tigecycline and lymecycline
	Macrolides	Bind to the 23S rRNA of the 50S ribosomal subunit, leading to incomplete peptide chains (Vázquez-Laslop and Mankin, 2018).	Azithromicin, erythromycin and clarithromicyn
	Lincosamides	Bind to the 50S ribosome subunit, causing the peptidyl-tRNA molecule to detach from the ribosome during elongation (Tenson et al., 2003).	Clindamycin
	Oxazolidinones	Inhibit the correct 70S ribosome subunit formation by binding to the 23S rRNA of the 50S subunit (Swaney et al., 1998).	Linezolid
DNA synthesis	Fluoroquinoles	Target DNA gyrase and topoisomerase IV inhibiting DNA replication (Correia et al., 2017; Ojkic et al., 2020).	Ciprofloxacin and levofloxacin
	Sulfonamides	Competitive inhibitor of Dihydropteroate synthase (DHPS) involved in folate synthesis (Wong et al., 2012).	Sulfamethazine, sulfapyridine
RNA synthesis	Rifamycins	Bind to the RNA polymerase and block the RNA synthesis (Kohanski et al., 2010).	Rifapentine, Rifampin

Examples of each antibiotic type are included.

* For more detailed classifications based on the antibiotic targets see O’Rourke et al. (2020), Wong et al. (2012), and Kohanski et al. (2010) for classifications based on chemical structure, see WHO (2023).

Traditionally, the pathway inhibited by a compound has been identified mainly through macromolecular synthesis (MMS) assays. These assays use radioactively labeled precursors for peptidoglycan, lipid, protein, RNA, or DNA synthesis (Cotsonas King and Wu, 2009), enabling the identification of whether one or more pathways are targeted. However, compounds that act on different stages of the same pathway cannot be identified. Despite being an important technique, MMS assays are limited by low accuracy, low resolution, low throughput and time-consuming (Nonejuie et al., 2013).

To address the limitations associated with MMS assays, diverse alternative techniques to identify the MOA have been developed (Silver, 2011; Da Cunha et al., 2021). These include biochemical approaches, that can start with molecular docking—a computational technique used to predict how a small molecule, such as an antibiotic, binds to its target (Fan et al., 2019). A complementary approach is affinity chromatography, which identifies direct biophysical interactions between antimicrobials and their targets where the antibiotic interacts with protein from whole-cell extracts (Hudson and Lockless, 2022; Hage et al., 2012; Franken et al., 2015). However, this requires a large amount of the test compound, which is often unavailable, particularly during the early stages of drug discovery (Nonejuie et al., 2013).

Identification of the molecular target can be achieved by employing genetic approaches (Cacace et al., 2017), such as resistance selection (Hudson and Lockless, 2022), testing specifically designed indicator strains or genetically modified mutant strains (Freiberg et al., 2005; Zlitni et al., 2013; Donald et al., 2009) and using pattern recognition techniques based on metabolomics (Vincent et al., 2016; Ribeiro da Cunha et al., 2020), such as Nuclear Magnetic Resonance Spectroscopy (Dörries et al., 2014; Hoerr et al., 2016) or Mass Spectrometry (Zampieri et al., 2018), as well as transcriptomics (Boshoff et al., 2004) through methods like hybridization assays (Boshoff et al., 2004) or next-generation sequencing (O’Rourke et al., 2020) and proteomics (Bandow and Hecker, 2007; Bandow et al., 2003). These approaches could be used independently or in conjunction with BCP to provide insights into antibiotic targets.

High-throughput screening platforms have been compared (Zampieri et al., 2018; Ayon, 2023) including some alternative discovery strategies (Quinn and Dyson, 2024). While these additional methods offer several benefits, they face significant limitations, particularly their time-consuming nature, which impacts their overall effectiveness. High cost and technical expertise are shared drawbacks of metabolomics, proteomics, and transcriptomics, as these methods rely on sophisticated equipment and complex data analysis. Similarly, transcriptional profiling and genetic approaches share the limitation of being unable to directly pinpoint molecular targets (Nonejuie et al., 2013). Apart from all limitations in determining MOA, discovering novel compounds that are active against Gram-positive and Gram-negative bacteria remains challenging.

The process of discovering and developing new classes of antibiotics is particularly challenging, as they must exhibit acceptable pharmacokinetic properties, demonstrate safety, and efficacy (Tacconelli et al., 2018). Moreover, producing antibiotics offers limited profit margins due to the high production costs and the extended process of research, testing, and approval (Tacconelli et al., 2018). Therefore, new, high-throughput screening platforms are needed for the fast and inexpensive screening of large libraries of synthetic and natural compounds that are highly effective against human pathogens (Lewis, 2013; Lewis et al., 2024). The following section reviews the quick and scalable bacterial cytological profiling methods (BCPs) and discusses their availability for some of the most important human pathogens as outlined in the latest WHO 2024 report.

## 3 BCP to identify the mechanism of action

**Figure 2:**
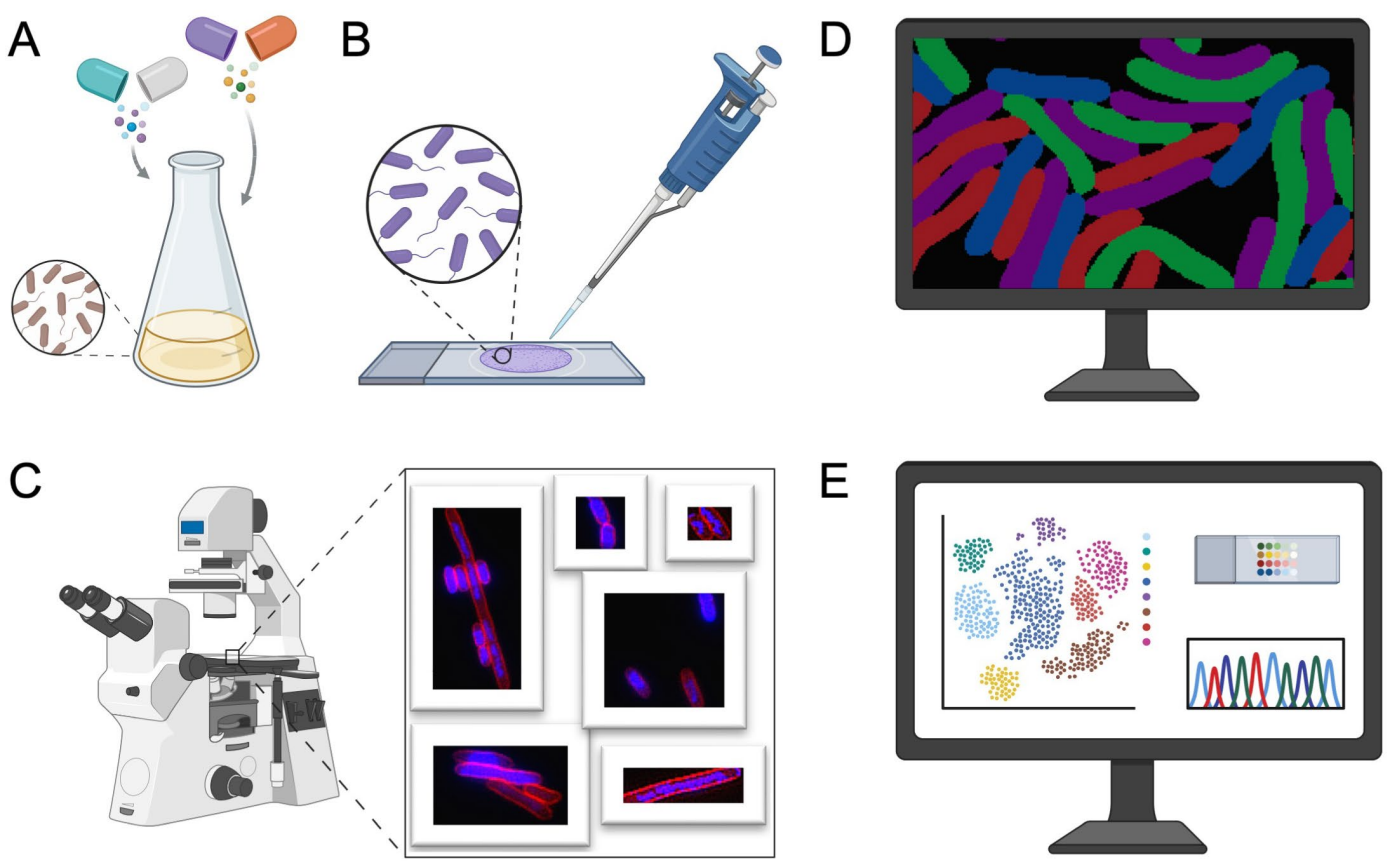
Bacterial cytological profiling workflow. **(A)** Bacterial culture preparation: cells are treated with antibiotics at specific concentrations. After incubation, cells are stained with fluorescent dyes targeting cellular components, such as the membrane and DNA. **(B)** Microscopy slide preparation. **(C)** Fluorescence microscopy is used to image antibiotic-treated cells. **(D)** Bacterial segmentation and image analysis, the resulting micrographs are processed using image analysis software to extract quantitative cytological data. **(E)** Extracted data is traditionally analyzed using Principal Component Analysis (PCA), see Box 1, to identify phenotypic changes, providing insights into the antibiotics’ mechanism of action (MOA). Figure is created using BioRender.

In 2013, Poochit et al. designed Bacterial Cytological Profiling (BCP) analysis for *E. coli* cells using different classes of antibiotics (Nonejuie et al., 2013) (Figure 2). BCP data are obtained using fluorescent microscopy of *E. coli* cells stained with fluorescent membrane and DNA dyes as well as fluorescent reporter for membrane permeability. Using image analysis software, various bacterial cell parameters, such as cell length, width, solidity, and DNA content, are extracted (Nonejuie et al., 2013). Subsequently, complex multidimensional data are analyzed using the *Principal Component Analysis (PCA)* technique, to cluster cells based on their cytological profile to identify the MOA of known and unknown antibacterial compounds (Figure 2; Box 1).

BOX 1 **Principal Component Analysis (PCA).**

**Figure 3:**
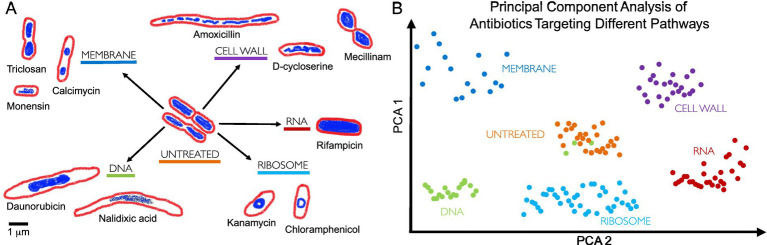
Bacterial cytological profiling. **(A)** Antibiotics that target different bacterial cell components induce different cell morphologies. The drawings are based on the microscopy images from Nonejuie et al. (2013), where bacterial cells were treated with antibiotics targeting five major biosynthetic pathways (DNA, Ribosome, RNA, Cell Wall, Membrane), using fluorescent dyes FM4-64 (red) and DAPI (blue) to stain bacterial membranes and DNA, respectively. Scale bar, 1 μm. **(B)** Principal Component Analysis (PCA) is used to cluster different bacterial cell shapes based on the antibiotic MOA, allowing profiles to be distinguished qualitatively. Each point on the graph represents a single cell, with clusters forming distinct groups according to cell morphology induced by different antibiotic treatments. For example, green dots represent cells treated with a DNA-targeting antibiotic, but we can see that some green dots cluster with orange dots (untreated bacteria), this suggests no significant morphological changes, indicating potential antibiotic resistance or persistence (Balaban et al., 2019; Kussell et al., 2005). PCA highlights MOA differences by visualizing the morphological variability induced by different antibiotic treatments.

PCA is a popular statistical technique commonly used for identifying linear relationships in complex data, such as those generated in BCP, by identifying the fewest variables which contribute the most to variance in the data (Bailey, 2012). PCA creates a linear combination of the original variables to create a new set of principal components (Bailey, 2012). Firstly, PCA starts by calculating a matrix showing the relation each variable has for all others. Then, it finds their eigenvectors (direction of the relationship) and eigenvalues (contribution of relationship to variance). In PCA, the principal components with the most contribution to variance in the data are then plotted. PCA has diverse applications across almost all scientific fields, including biology, medicine, computer science, and geology. In the context of biomedical research, PCA has been utilized to analyze the human cell atlas and prostate cancer risk prediction (van et al., 2014; Regev et al., 2017). Whilst PCA captures linear relationships between variables exclusively, it has been the primary method for dimensional reduction in Bacterial Cytological Profiling (Figure 3B). There are also recent popular approaches capturing non-linear relationships such as UMAP (McInnes et al., 2020) (primarily local differences), or PaCMAP (Wang et al., 2021) (local and global relationships) which may provide other insights into local differences and non-linear relationships within and between antibiotic treatments. Additionally, plots of dimensionally reduced provide insightful comparisons between different treatments, however, identifying distinct profiles is not equivalent to identifying novel targets; a drug could have multiple known MOAs for example and still display a distinct profile (Samernate et al., 2023; Martin et al., 2020). Even so, there have been possible connections found between various BCP profiles and novel targets using PaCMAP (Takebayashi et al., 2024).

Since cytological profiling produces data at a single-cell level (Samernate et al., 2023), it uses morphological data such as bacterial chromosomal condensation, or cell shape changes in response to antimicrobials to differentiate between different targeted metabolic pathways (Nonejuie et al., 2013) (Figure 3). Furthermore, this approach can lead to the identification of antibiotics that are effective against multidrug-resistant bacteria (Quach et al., 2016).

BCP takes advantage of the limited presence of cell-cycle checkpoints in bacteria (Nonejuie et al., 2013). When stressed by antibiotics, bacteria show phenotypical changes that are characteristic of the antibiotic target. For example, compounds that target the ribosome by stopping protein synthesis (e.g., tetracycline and chloramphenicol) produce circular chromosomes and wide cells (Nonejuie et al., 2013; Wu et al., 2019) (Figure 3).

During antibacterial treatment, rod-shaped bacteria (bacilli) can shrink and take on an oval form, known as ovoid cells (Spratt, 1975; Spratt and Pardee, 1975). There are no clearly defined names for these cells, however, as they have been referred to in literature as ‘round forms’ (Curtis et al., 1979; di et al., 1994); ‘round cells’ (Bernabeu-Wittel et al., 2004; Jackson and Kropp, 1996; de Pedro et al., 2001); ‘spherical forms’; ‘spherical cells’ (Sumita et al., 1990; Dalhoff et al., 2003; Horii et al., 1998); or ‘coccoid forms’ (Perumalsamy et al., 2013; Nickerson and Webb, 1956). Filamentation, or cell elongation, occurs when rod-shaped bacteria (or sometimes cocci) synthesize peptidoglycan for their side walls but not for their division walls, leading to abnormally elongated cells (Cushnie et al., 2016). This process results from the inhibition of septal peptidoglycan synthesis (Spratt, 1975). Filamentous cells can be also induced when DNA synthesis is inhibited (Elliott et al., 1987; Chen et al., 2005) or DNA is damaged (Uphoff et al., 2013; Jones and Uphoff, 2021; Jaramillo-Riveri et al., 2022) by a process known as the SOS response that inhibits cell division (Ojkic et al., 2020) (Figure 3).

Antibiotic treatments can drastically alter bacterial cell size, induce localized swelling, bulge formation, blebbing, and thicken peptidoglycan (Cushnie et al., 2016). Occasionally, antibiotic-treated cells can lose cell walls, turning bacterial cells into spheroplasts and protoplasts. Spheroplast are Gram-negative bacteria that lost their peptidoglycan layer, but kept their outer membrane, whereas protoplasts are formed from Gram-positive bacteria that lack the peptidoglycan layer (Gebicki and James, 1960; Ojkic et al., 2014). Bacterial variants that completely lack a cell wall, encompassing both Gram-negative and Gram-positive bacteria, are also known as *L-forms* (Errington, 2013; Allan et al., 2009; Mercier et al., 2014; Errington, 2017).

Phenotypical changes derived from the antibiotic-induced changes in bacterial subcellular architecture could confer an increase in fitness to bacteria in the presence of antibiotics (Banerjee et al., 2021). Resistance to antibiotics usually takes the form of reducing the concentration of intracellular antibiotic or by reducing the binding affinities of the cellular targets to the antibiotic (Ojkic et al., 2022; Baquero et al., 2023). By using available BCP data, recent studies have shown that by reducing the surface-to-volume ratio (*S/V*), bacteria can effectively reduce the antibiotic concentration inside a cell, thereby promoting cell growth by decreasing antibiotic influx (Ojkic et al., 2022). Similarly, an increase in *S/V* can benefit the cell in alternative ways such as increasing the antibiotic efflux rate or the rate of nutrient uptake (Ojkic et al., 2022; Ojkic et al., 2019; Ojkic and Banerjee, 2021). These studies explain how cell shape transformations promote bacterial survival under antibiotic treatments—pointing toward potential new druggable targets that control cell shape and size under stress.

**Figure 4:**
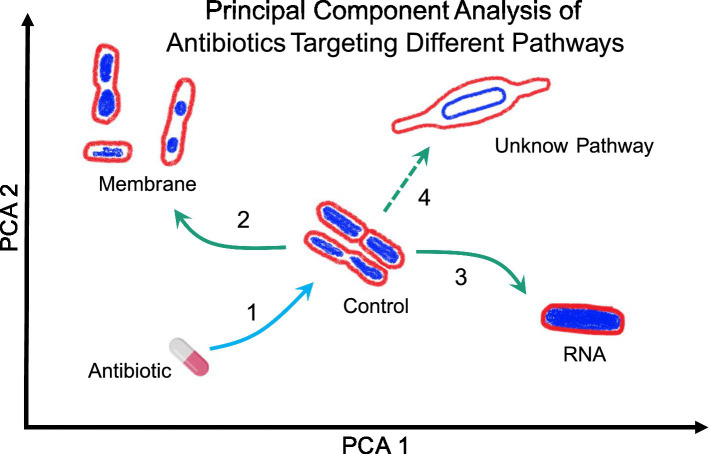
Representation of Principal Component Analysis (PCA) using bacterial morphologies to determine the MOA of a novel antibiotic. Arrow 1 indicates the antibiotic used against bacteria studied, which can change their shape depending on the antibiotic’s MOA. For example, if the bacteria exhibit a morphology as indicated by arrow 2, the antibiotic likely targets the membrane. Conversely, if the bacteria display a morphology as indicated by arrow 3, the antibiotic likely targets RNA. However, if the morphology is completely different from the known and clustered morphologies, as shown by arrow 4, it suggests that the antibiotic targets a novel pathway but lastly, if the bacteria do not show any change, it suggests that they are not susceptible to the tested antibiotic or, in the worst-case scenario, that they are resistant to said antibiotic.

BCP has been successfully employed to study the MOA of various antibacterial agents, including azithromycin (Lin et al., 2015), diphenylureas (Mohammad et al., 2017) and thailandamide (Wu and Seyedsayamdost, 2018). It has also been used to identify the cellular pathways targeted by anticancer metal complexes (Sun et al., 2018) and to study the response of bacteria to antibiotics in different growth conditions (Dillon et al., 2020). Additionally, BCP has been used to identify the cellular pathways targeted by antibacterial molecules affecting different cellular pathways (Araújo-Bazán et al., 2016; Andreu et al., 2022), making it a valuable tool not only for determining antibacterial targets but also to potentially identify novel MOA, i.e., ones that target new proteins or new pathways (Figure 4).

BCP can also be used to determine the MOA of treatments beyond antibiotics, such as phage therapies, revealing how phages disrupt essential cellular pathways (Deep et al., 2024; Tsunemoto et al., 2023; Birkholz et al., 2024; Thammatinna et al., 2020; Soonthonsrima et al., 2023; Naknaen et al., 2023; Naknaen et al., 2024). BCP allows the visualization of bacterial chromosomal condensation, cell shape and overall cellular morphology changes within bacterial cells during phage infection. These changes not only reveal the pathways and cellular targets phages use to propagate their lifecycle but also highlight the role of bacterial defense mechanisms in combating phage infection (Tsunemoto et al., 2023; Thammatinna et al., 2020; Naknaen et al., 2024). BCP has demonstrated how the overexpression of phage-related proteins can induce specific phenotypic changes as a result of the activation of a bacterial defense system to suppress phage propagation (Deep et al., 2024). Additionally, BCP has been fundamental in assessing the impact of different antibiotics on phage replication, revealing that certain antibiotics can synergize with phages to enhance bacterial cell lysis. In contrast, others inhibit phage propagation by disrupting essential bacterial processes (Tsunemoto et al., 2023). This dual capability of BCP to show both the direct effects of phage infection and the influence of external agents such as antibiotics, makes it a high-throughput tool in studying phage-bacteria dynamics.

## 4 BCP of important human pathogens

Most importantly, BCP has been successfully used to study some of the most important human pathogens from the WHO Bacterial Priority Pathogens List (Table 2). In 2017, the first Bacterial Priority Pathogen list was created by the WHO in collaboration with researchers from the Division of Infectious Diseases at the University of Tübingen, Germany which used a multicriteria analysis technique to inform research and development (R&D) for future antibacterial compounds (World Health Organization, 2017). Now, 7 years after the introduction of the list there have been novel antibiotics put onto the market either with effectiveness *in vivo* or *in vitro* against pathogens deemed critical priority, but unfortunately, resistance has been found in almost every one (di et al., 2021; Butler et al., 2022). This year the WHO updated this list to tackle new developments in antimicrobial resistance to give an updated and directions for policy makers and insight on future developments. The new Bacterial Priority Pathogens List 2024 includes 15 resistant pathogens, ordered at various levels of priority from medium; high; to critical (WHO Bacterial Priority Pathogens List, 2024) (Table 2). Out of 15 pathogen groups, bacterial cytological profiling is not available for 30% of them: *Non-typhoidal Salmonella, Neisseria gonorrhoeae*, Group A and B *Streptococci, Haemophilus influenzae*. Therefore, urgent BCPs regarding these severe pathogenic organisms are needed.

**Table 2:** BCP analysis studies checklist from the WHO Bacterial Priority Pathogens List (2024) separated by priority from 1 to 3.

Bacteria	Resistant to	Bacterial Cytological Profiling (BCP)
Priority 1. Critical group		
*Acinetobacter baumannii*	Carbapenems	Yes (Samernate et al., 2023; Lin et al., 2015; Htoo et al., 2019)
*Enterobacteriaceae* ^*^	Third generation cephalosporine	Yes (Nonejuie et al., 2013; Sun et al., 2018; Coram et al., 2022; Montaño et al., 2021; Nonejuie et al., 2016)
*Enterobacteriaceae* ^**^	Carbapenems, ESBL-producing	Yes (Lin et al., 2015; Sun et al., 2022; Sridhar et al., 2021)
*Rifampicin-Resistant Tuberculosis (RR-TB)* ^***^	Rifampicin	Yes (Smith et al., 2020; Allen et al., 2024)
Priority 2. High group		
*Salmonella Thypi*	Fluoroquinolones	Yes (Sridhar et al., 2021)
*Shigella* spp.	Fluoroquinolones	Yes (López-Jiménez et al., 2024)
*Enterococcus faecium*	Vancomycin	Yes (Werth et al., 2014)
*Pseudomonas aeruginosa*	Carbapenems	Yes (Tsunemoto et al., 2023; Lin et al., 2015; Sun et al., 2022; Sridhar et al., 2021)
*Non-typhoidal Salmonella*	Fluoroquinolones	No
*Neisseria gonorrhoeae*	Cephalosporin, Fluoroquinolones	No
*Staphylococcus aureus*	Methicillin and vancomycin	Yes (Quach et al., 2016; Mohammad et al., 2017; Kalla, 2020; Blaskovich et al., 2021)
Priority 3. Medium group		
Group A *Streptococci*	Macrolide	No
*Streptococcus pneumoniae*	Macrolide/No sensitivity to penicillin	Yes (Sakoulas et al., 2015)
*Haemophilus influenzae*	Ampicillin	No
*Group B Streptococci*	Penicillin	No

First column, the category of bacteria; second column, antibiotic that bacterial group is resistant to; third column indicates whether bacteria have been studied using Bacterial Cytological Profiling (BCP) or not. We consider any BCP done in wild-type strains rather than AMR pathogen strains.

* The BCP column uses *Escherichia coli* as a reference for this group.

** The BCP column uses *Klebsiella pneumoniae* as a reference for this group.

*** RR-TB was evaluated independently in a tailored approach so it was technically “not” included in the list but after the evaluation by specialists, it was determined as a critically dangerous bacteria therefore. RR-TB stands apart from the list due to the distinct nature of its evaluation process.

## 5 BCP to identify new druggable cell pathways

BCP is used to scan new antibacterial components to identify their specific targets (Figure 4). As demonstrated, BCP effectively differentiates between various morphological changes induced by different antibiotics, thereby providing insights into the antibiotic’s MOA. If a novel antibiotic places bacteria in a distinct region of the PCA plot compared to known antibiotic targets, it could indicate a new pathway target or MOA previously uncharacterized (Figure 4). For example, if the PCA analysis shows that the morphology of bacteria treated with a new antibiotic clusters in a region associated with membrane or RNA targets (Arrows 2 and 3 in Figure 4), it directly indicates the antibiotic’s mode of action. Conversely, if the antibiotic’s effect causes a morphology change that places bacteria in a novel zone, as illustrated with Arrow 4, it may suggest the discovery of a new antibacterial pathway or target.

Together, BCP significantly enhances drug development by offering a precise, fast and systematic method for characterizing the effects of new antibacterial agents. Its ability to identify target-specific morphological changes provides a comprehensive tool for uncovering novel antibiotic targets and advancing our understanding of bacterial physiology.

## 6 Image analysis tools for BCP and data availability

Fluorescent microscopy has allowed us to visualize many cell components in great clarity, however, quantitative image analysis of both cellular and sub-cellular structures has been a continual challenge (Mistretta et al., 2024; Ma et al., 2024). The requirement for accurate tools is highlighted by the scale of the bacterial objects, with typically 100–300 pixels per typical *E. coli* cell (Cutler et al., 2022), while antibiotic-treated cells could have an order of magnitude larger sizes (Nonejuie et al., 2013). In addition to variations in size, bacteria exhibit a diverse array of shapes (Young, 2006). While most studied bacteria in BCP (see Tables 3, 4) are rod-shaped or spheroid, there is growing interest in bacteria with more complex shapes that emerge after exposure to antibiotics, such as seen in *Caulobacter crescentus* (Banerjee et al., 2021).

**Table 3:** Studies with bacterial profiles following the original BCP method, that being the use of segmentation, feature extraction, and dimensional reduction techniques to create a plot allowing the viewer to differentiate between different antibiotic treatments on the same bacterial cell strains.

Organism	Dyes/Fluorophores	Processed data available	Segmentation	Feature extraction	Source
*Acinetobacter baumannii* and *E. coli*	FM4-64 DAPI SYTOX-Green	Yes	CellProfiler (McQuin et al., 2018)	CellProfiler (McQuin et al., 2018)	Htoo et al. (2019)
*Acinetobacter baumannii*	FM4-64 DAPI SYTOX-Green	No	Ilastic (Berg et al., 2019)	CellProfiler (McQuin et al., 2018)	Samernate et al. (2023) ^*^
*Pseudomonas aeruginosa*	FM4-64 DAPI	Yes	Manually (FIJI/ImageJ (Schindelin et al., 2012))	Manually (FIJI/ImageJ (Schindelin et al., 2012))	Tsunemoto et al. (2023) ^*^
*S. aureus*	FM4-64 DAPI SYTOX-Green	Yes	Semi-Manual (FIJI/ImageJ (Schindelin et al., 2012))	Semi-Manual (FIJI/ImageJ (Schindelin et al., 2012))	Nonejuie et al. (2013) ^*^
*S. aureus*	FM4-64 DAPI SYTOX-Green WGA-647	Yes	CellProfiler (McQuin et al., 2018)	CellProfiler (McQuin et al., 2018)	Quach et al. (2016) ^*^
*S. aureus*, *S. typhimurium*, and *K. pneumoniae*	FM4-64 DAPI SYTOX-Green	Yes	Harmony (Korsunsky et al., 2019)	Harmony (Korsunsky et al., 2019)	Sridhar et al. (2021)
*B. subtilis*	FM 4–64 DAPI SYTOX Green	Yes	CellProfiler (McQuin et al., 2018)	CellProfiler (McQuin et al., 2018) FIJI	Lamsa et al. (2016) ^*^
*B. subtilis*	FM4-64 DAPI SYTOX-Green	No	CellProfiler (McQuin et al., 2018)	CellProfiler (McQuin et al., 2018)	Herschede et al. (2022) ^*^
*B. subtilis*	Nile red DAPI	No	MicrobeJ (Ducret et al., 2016)	MicrobeJ (Ducret et al., 2016)	Schäfer et al. (2024) ^*^
*Bacillus subtilis*, *E. coli*	FM4-64 DAPI GFP	No	Wasabi software (Hamamatsu)	Wasabi software (Hamamatsu)	Araújo-Bazán et al. (2016)
*E. coli*	FM4-64 Hoechst-33342 Dendra2 protein	No	FIJI/ImageJ (Schindelin et al., 2012)	FIJI/ImageJ (Schindelin et al., 2012)	Sun et al. (2018)
*E. coli*	FM4-64 DAPI SYTOX-Green	No	Semi-Manual (FIJI/ImageJ (Schindelin et al., 2012))	Semi-Manual (FIJI/ImageJ (Schindelin et al., 2012))	Montaño et al. (2021) ^*^
*M. tuberculosis* Erdman	FM4-64FX SYTO 24	Yes	MorphEUS (Hausen and Robertson, 2020)	MorphEUS (Hausen and Robertson, 2020)	Sun et al. (2022) ^*^

This table includes the organisms studied, the dyes used to visualize cellular components, data availability, and image analysis methods used to extract data for profiling.

* Pipelines, scripts, or instructions are detailed and/or included in the paper. Programs are also widely accessible.

**Table 4:** Studies using protocols similar to the original BCP method by merit of developing a profile or differentiating between bacterial cells which exhibit different phenotypes.

Organism	Dyes/Fluorophores	Processed data available	Segmentation	Feature extraction	Source
*E. coli* *Caulobacter crescentus*	FM4-64 DAPI	No	Oufti (Paintdakhi et al., 2016)	Oufti (Paintdakhi et al., 2016)	Santos et al. (2018) ^*^
*Achromobacter xylosoxidans*	FM4-64 DAPI SYTOX-Green NBD Azithromycin	No	FIJI/ImageJ (Schindelin et al., 2012) and CellProfiler (McQuin et al., 2018)	FIJI/ImageJ (Schindelin et al., 2012) and CellProfiler (McQuin et al., 2018)	Ulloa et al. (2020)
*M. smegmatis*	ParB-mCherry	Yes	MicrobeJ (Ducret et al., 2016)	MicrobeJ (Ducret et al., 2016)	de Wet et al. (2020) ^*^
*Shewanella putrefaciens*	Ffh-mVenus FtsY-mVenus uL1-mVenus	Yes	FIJI/ImageJ (Schindelin et al., 2012)	FIJI/ImageJ (Schindelin et al., 2012)	Mayer et al. (2022) ^*^
*V. parahaemolyticus*	FM4-64 DAPI	No	FIJI/ImageJ (Schindelin et al., 2012)	FIJI/ImageJ (Schindelin et al., 2012)	Soonthonsrima et al. (2023)
*Bacillus subtilis*	FM4-64 DAPI SYTOX-Green SYTO-9	No	–	Manual w/ FIJI (Schindelin et al., 2012)	Ouyang et al. (2022) ^*^
*M. smegmatis* *M. tuberculosis*	FM4-64 GFP CellROX	Yes	Omnipose (Cutler et al., 2022)	Manual w/ FIJI or with Cell Counter installation in FIJI Custom python script	Mistretta et al. (2024)
*E. coli* *B. subtilis*	FM4-64 DAPI GFP DiSC	No	FIJI/ImageJ (Schindelin et al., 2012) and MicrobeJ (Ducret et al., 2016)	FIJI/ImageJ (Schindelin et al., 2012) and MicrobeJ (Ducret et al., 2016)	El-sagheir et al. (2023)
*E. coli* *S. aureus* *Bacillus subtilis*	Nile Red DAPI mScarlet	Yes	StarDist (Weigert and Schmidt, 2022) CARE (Weigert et al., 2018) pix2pix (Isola et al., 2018) ML-U-Net (Feng et al., 2024) SplineDist (Mandal and Uhlmann, 2021)	Classification without feature extraction, using Deep Learning	Spahn et al. (2022)

This table includes the organisms studied, the dyes used to visualize cellular components, whether the data is available online, and image analysis methods used to extract data for profiling.

* Pipelines, scripts, or instructions are detailed and/or included in the paper. Programs are also widely accessible.

To precisely define cell boundaries and to segment cellular components, sub-pixel segmentation methods are required (Cutler et al., 2022; Bali and Singh, 2015). Classical image segmentation techniques have been used since the 1960s (Prewitt and Mendelsohn, 1966; Mendelsohn et al., 1965), laying the groundwork for the more advanced artificial intelligence methods used nowadays. Many segmentation solutions are currently available as user-friendly plugins such as MicrobeJ (Ducret et al., 2016) within easily accessible platforms such as ImageJ (Ma et al., 2024; Stringer et al., 2021; Sousa, 2020). MicrobJ is based on the classical segmentation method, but since December 2024, MicrobJ has implemented the deep neural network segmentation algorithm Omnipose. Stand-alone image analysis programs are also available but these can at times be less supported and less accessible (Paintdakhi et al., 2016).

In 2016 SuperSegger was created to improve upon flaws in segmentation through thresholding in bacterial phase-contrast images and it combines classical segmentation with Deep learning (Stylianidou et al., 2016). To correct common errors in segmentation from both the thresholding and watershed, SuperSegger uses a shallow neural network trained from the segmentation data (Chai et al., 2023). Recently, Deep neural networks (DNNs) which have now become the backbone of most Deep learning segmentation methods are now widely recognized as superior tools for cell segmentation (Jan et al., 2024). As showcased in Table 3, Deep learning is significantly underutilized in original BCP studies. However, some recent BCP-like profiles have been created using deep learning for segmentation and profiling (Zagajewski et al., 2023), and object detection (Spahn et al., 2022) for antibiotic susceptibility analysis (Table 4).

Among recent and easily available Deep learning segmentation algorithms, Pachitariu et al. demonstrated that Cellpose outperformed the popular programs Mask R-CNN and StarDist when applied to a varied dataset of different cell types and cell-like-objects, showcasing it as a powerful general solution for cell segmentation (Stringer et al., 2021). Cutler et al. assessed the performance of (at the time) state-of-the-art cell segmentation algorithms on a wide array of bioimages of bacteria with different morphologies. This led to the design of the algorithm, *Omnipose*, which outperformed all segmentation algorithms tested across a varied dataset of bacterial cell sizes, shapes, and optical characteristics and as such has been used extensively in research (Cutler et al., 2022). Recent benchmarks for bacterial segmentation algorithms have found that transformer based algorithms currently outperform other deep learning models such as CNNs, and classical segmentation techniques on a multitude of cell types when well implemented on a large datasets (Ma et al., 2024). The major advantages these algorithms have over their competing peers are threefold: They have a larger model capacity, allowing them to train for a more complex task than a model with lower capacity; are able to find patterns over the whole image due to self-attention mechanisms when CNNs are only able to do so in smaller regions of the image (Vaswani et al., 2023); less annotation is needed than competing algorithms for large datasets (Ma et al., 2024) as the model can pretrain using transfer-learning and fine tune with more limited annotations. The top three winning algorithms of the multimodality cell segmentation challenge have however not been used for BCP despite their recent inclusion in the widely accessible program, NAPARI (Sofroniew et al., 2025).

Segmented data availability (Tables 3, 4) is invaluable for scientific communities and accelerates new findings by increasing the reproducibility of datasets and enabling future meta-analyses. By using published BCP data and mathematical modeling, the researchers uncovered the robustness of scaling behavior between cell surface area and volume in *E. coli* (Ojkic et al., 2019) and B. subtilis (Ojkic and Banerjee, 2021), inferred cell physiological alterations upon antibiotic treatments (Cylke et al., 2022), and proposed a new antibiotic resistance pathway mediated by cell surface-to-volume ratio (*S/V*) transformations (Ojkic et al., 2022). Therefore, the availability of BCP data is good practice and should be considered as a benchmark for all future BCP platforms, especially for pathogenic bacteria (Table 2).

## 7 BCP limitations

Even with all the advantages we mentioned about BCP, it has certain limitations. BCP can identify the general target of an antibiotic, but it cannot provide precise information about the exact site within the target that is affected. For instance, while BCP can indicate that an antibiotic targets the ribosome, it cannot specify which part of the ribosome is involved. Other high-throughput methods, such as affinity chromatography and omics-based approaches, can predict cellular targets more accurately by providing detailed insights into specific molecular interactions or pathway alterations. Combining these techniques with BCP could help overcome BCP’s limitation in pinpointing the exact stage of pathway inhibition.

BCP requires staining dyes to evaluate DNA content and cell size and shape, with fluorescent dye intensity being essential for determining the antibiotic MOA. A wide variety of dyes, protein fusions, and reporter strains have been used in BCP, facilitating both fast MOA detection, and discovery of new MOAs. However, despite the abundance of possible dyes, strains, and assays selecting the most appropriate ones for specific phenotypic experiments remains challenging, as more information is needed to understand cellular functions (Table 3). This is exacerbated by the complexity of bacterial physiology with many processes being overlapped by mechanisms such as metabolic flux or co-dependent regulation (Schäfer et al., 2024; Serbanescu et al., 2022). While numerous cytological profiles have been reported, neither antibiotic concentration nor time of antibiotic exposure has been standardized. Additionally, scaling BCP in low-resource settings may be challenging, particularly due to the reliance on specialized microscopes and fluorescent dyes.

## 8 BCP potential improvements

As shown in Tables 3, 4, a variety of fluorescent dyes have been used to investigate the cytological profiles of different bacterial organisms. However, newly developed dyes have the potential to provide more detailed information that could help in building a more comprehensive response profile. Despite the prevalence of cell wall targeting antibiotics in BCP experiments, direct methods for visualizing cell wall synthesis and remodeling during antibiotic exposure have been lacking. In 2012, Kuru et al. discovered a groundbreaking method for bacterial cell wall staining using fluorescent amino acids (Kuru et al., 2012). The cell wall provides the shape and structural integrity of the cell. It is made of peptidoglycan (PG), which consists of glycan strands cross-linked by D-amino acid (DAA) (Vollmer et al., 2008). The team introduced HADA and NADA, two fluorescent D-amino acids (FDAAs) attached to a D-amino acid backbone (3-amino-d-alanine). This chemical biology approach aims to detect and visualize the exact location and amount of new peptidoglycan layer synthesis in bacteria. By using HADA or NADA as a fluorescent peptidoglycan label during cell wall synthesis, the technique also allows researchers to observe morphological changes in bacteria over time. This is relevant as HADA and NADA can be implemented in the methodology of BCP to investigate the growth modes of bacteria under antibiotic exposure as they exhibit a diverse growth pattern—that could confer selective advantages in their environments (Young, 2006; Ojkic et al., 2022).

Other fluorescent dyes are available to quantitatively probe bacterial physiological states: ThT and DiBAC_4_ for bacterial membrane potential (Stratford et al., 2019; Prindle et al., 2015; De Souza-Guerreiro et al., 2024; Wong et al., 2021), carboxy-H_2_DCFDA for reactive oxygen species (ROS) (Wong et al., 2021), and DAF-FM for reactive nitrogen species (RNS) (Wong et al., 2021). By integrating membrane potential, ROS, and RNS into cytological profiles could provide additional information regarding bacterial physiology and bacterial stress response during antibiotic treatment. However, adding additional dyes could be a challenge in low resource settings. With additional dyes also comes increasing potential problems with fluorescence crosstalk. Fortunately, there have been recent advances in AI image analysis which could circumvent this problem by predicting and generating overlays of fluorescent dyes onto unlabeled cells (Goldsborough et al., 2017; Osokin et al., 2017).

## 9 Conclusion

Despite significant advances in research and the development of new tools, combating antimicrobial resistance (AMR) requires a multifaceted approach. Continued investment in research and development, global collaboration, and the effective implementation of surveillance and prevention strategies are crucial. Bacterial Cytological Profiling (BCP) stands out as a rapid and cost-effective technique that facilitates drug discovery by revealing the mechanism of action of novel antibacterial agents through detailed physiological and morphological analysis. Furthermore, BCP could be used to identify phenotypic changes when multiple antibiotics are used, revealing unique or overlapping cell morphologies induced by these combinations (Samernate et al., 2023). However, systematic explorations of cytological profiles for drug combinations are still missing.

Apart from bacteria, cytological profiling methods are also widely used for other organisms such as yeast (Chessel and Carazo Salas, 2019; Chong et al., 2015), fungi (McMahon et al., 2023), and human cells (McDiarmid et al., 2024; Ren et al., 2021; Perlman et al., 2004). Deep learning techniques employed for yeast and human cells have been used without feature extraction, however, this method has only recently been used for bacteria (Spahn et al., 2022). Therefore, wider availability, applications and integration of machine learning tools across different scientific fields are needed.

Besides BCP being used to discover new antibiotics, BCP has been used to investigate complex interactions between bacteria and their predators—bacteriophages (Deep et al., 2024; Tsunemoto et al., 2023; Birkholz et al., 2024). BCP enables the identification of metabolic pathways and cellular processes targeted by phages and antibiotics, both individually and in combination. Therefore, BCP reveals molecular mechanisms governing the phage-bacteria interaction, ultimately paving the way for more effective phage-based antibacterial therapies (Thompson et al., 2024; Kunisch et al., 2024). While bacteriophages have been used in BCP studies, intracellular pathogens present a future challenge as these cytological profiles may strongly depend on host-specific interactions.
